# From Structure to Function: Analysis of the First Monomeric Pyridoxal-5′-Phosphate-Dependent Transaminase from the Bacterium *Desulfobacula toluolica*

**DOI:** 10.3390/biom14121591

**Published:** 2024-12-12

**Authors:** Alina K. Bakunova, Ilya O. Matyuta, Alena Y. Nikolaeva, Tatiana V. Rakitina, Konstantin M. Boyko, Vladimir O. Popov, Ekaterina Yu. Bezsudnova

**Affiliations:** 1 Bach Institute of Biochemistry, Research Centre of Biotechnology of the Russian Academy of Sciences, Leninsky Ave. 33, bld. 2, 119071 Moscow, Russia; a.bakunova@fbras.ru (A.K.B.); i.matyuta@fbras.ru (I.O.M.); aishome@mail.ru (A.Y.N.); kmb@inbi.ras.ru (K.M.B.); v.popov@fbras.ru (V.O.P.); 2 Moscow Center for Advanced Studies, Kulakova Str. 20, 123592 Moscow, Russia; 3 Shemyakin & Ovchinnikov Institute of Bioorganic Chemistry RAS, Miklukho-Maklaya Str. 16/10, 117997 Moscow, Russia; taniarakitina@yahoo.com; 4 Biological Department, Lomonosov Moscow State University, Leninskiye Gory 1/3, 119991 Moscow, Russia

**Keywords:** pyridoxal-5′-phosphate, D-amino acid, transaminase, monomer, enzyme catalysis, X-ray analysis, structure–function relationships

## Abstract

The first monomeric pyridoxal-5′-phosphate (PLP)-dependent transaminase from a marine, aromatic-compound-degrading, sulfate-reducing bacterium *Desulfobacula toluolica* Tol2, has been studied using structural, kinetic, and spectral methods. The monomeric organization of the transaminase was confirmed by both gel filtration and crystallography. The PLP-dependent transaminase is of the fold type IV and deaminates D-alanine and (*R*)-phenylethylamine in half-reactions. The enzyme shows high stereoselectivity; no deamination of L-amino acids and (*S*)-phenylethylamine is detected. Structural analysis and subsequent mutagenesis led to the conclusion that the monomeric architecture of the enzyme is the only one possible and sufficient for stereoselectivity and PLP binding, but not for the overall double-substrate transamination reaction and the stability of the holo form with the reduced cofactor—pyridoxamine-5′-phosphate. These results extend the structural university of the PLP fold type IV enzymes and demonstrate the need for deeper analysis of the sequence–structure–function relationships in the transaminases.

## 1. Introduction

The oligomeric composition of enzymes is crucial for their catalytic function, stability, and activity. Among pyridoxal-5′-phosphate (PLP)-dependent enzymes, the most common functional unit is a dimer or multiple dimer (tetramer of hexamer) [1,2]. PLP-dependent enzymes catalyze a wide variety of reactions (transamination, racemization, decarboxylation, retro-aldol condensation, elimination, etc.) [2,3]. Five (I-V) fold types of PLP-dependent enzymes are known today, and the dimer is the minimal assembly required for catalysis among the different fold types [1,4,5,6]. Structural analysis of PLP-dependent enzymes from different fold types reveals the involvement of residues from both subunits in PLP binding and substrate accommodation in two symmetrical active sites, which are located at the dimer interface [2,4,7,8]. Nevertheless, the structural diversity is not limited to these observations. For example, the active site of the L-threonine aldolase tetramer from *Escherichia coli* contains residues from three subunits [9]. Next, the fold II enzymes operate in dimeric, tetrameric, or oligomeric forms, with the active site being organized by residues from one subunit, and many fold type II enzymes are under allosteric regulation [10]. Yeast threonine synthase of fold type II is a monomer with the PLP binding site buried deeply within a wide cleft that penetrates the whole molecule [11]. Therefore, the widely observed binding of PLP and substrates by the residues of both subunits of the dimer is not a prerequisite for catalysis.

**Figure 1:**
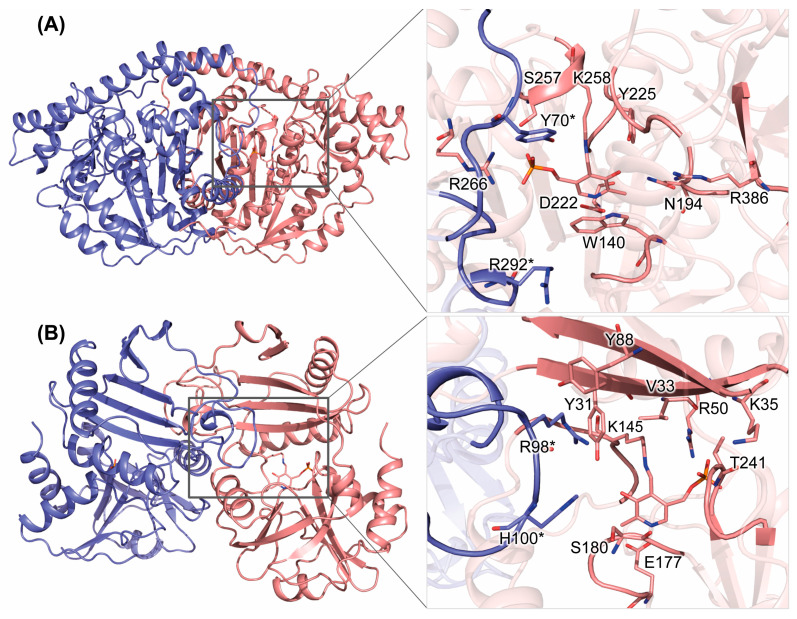
The organization of the active sites in transaminases. (**A**) The crystal structure of PLP fold type I aspartate aminotransferase from *E. coli* (PDB ID: 1ARS) with a zoomed-in view of the active sites. (**B**) The crystal structure PLP fold type IV D-amino acid transaminase from *Bacillus* sp. YM-1 (PDB ID: 4DAA) with a zoomed-in view of the active site. One subunit is colored in pink and the adjacent subunit is in blue. Residues of the adjacent subunit are marked (*).

In PLP-dependent transaminases (aminotransferases) of I and IV fold types, the dimer is a functional unit, with the active site formed by the residues of both subunits [2,12,13,14] (Figure 1). No monomeric transaminase was described. Dimer dissociation induces inactivation of transaminase [15]. Some tetrameric and hexameric transaminases are found in archaea, thermophilic, and mesophilic bacteria (e.g., branched-chain amino acid transaminase (BCAT) from *E. coli* and (*R*)-amine pyruvate transaminase (R-ATA) from *Arthrobacter* sp. KNK168) [14,16,17]. While in hexameric transaminases the active site is organized by the residues of the neighboring subunits forming the functional dimer, the multiple dimer organization may be a stabilizing factor or a consequence of the surface properties of the subunits [14,17,18,19].

The enzymatic transamination, regardless of the substrates involved in the reaction, is a double-displacement process. The first half-reaction is the stereospecific transfer of an amino group from amino acid or amine to the PLP cofactor through an external aldimine, a quinonoid intermediate, a ketimine, and a carbinolamine to give keto acid or ketone and pyridoxamine 5′-phosphate (PMP). In the second half-reaction, PMP is converted to PLP via the stereospecific transfer of an amino group to the second substrate, keto acid, or ketone, through the same intermediates in reverse order to produce a new amino acid or amine [2,3,20]. Stereoselectivity, substrate specificity, and catalytic efficiency of the catalyzed transaminase reactions are determined by the amino acid composition of the active site.

The subunit of the fold type I homodimer of transaminases contains two domains: the large cofactor-binding domain has a seven-stranded parallel β-sheet surrounded by seven α-helices; the small domain above the active site has residues from the N- and C-termini. PLP is located in a cleft between these domains. There are two active sites at the dimer interface and each subunit contributes residues to both. In aspartate aminotransferase, the large and small domains move significantly upon association with the substrate, forming a closed conformation that is important for specificity and reaction type [2,6,7,21].

The subunit of the fold type IV homodimer of transaminases contains two domains: the large domain represents a C-terminal six-stranded β-barrel core; the N-terminal small domain is connected to the large domain by the interdomain loop. There are two active sites at the dimer interface, with the PLP being bound by residues from both subunits. The active site of type IV transaminases is well described as a combination of two pockets: the O-pocket on the O-side of the PLP and the P-pocket on the P-side of the PLP. The O-pocket and interdomain loops cover the active site in some transaminases. These loops are critical for substrate binding [14,22,23,24,25,26]. In some substrate-free (unliganded) structures, both the interdomain and O-pocket loops are disordered; however, they are well defined in complexes with substrate [13,14,17,23,27,28].

Here, we describe a monomeric PLP-dependent fold type IV transaminase found in a marine aromatic-compound-degrading sulfate-reducing bacterium *Desulfobacula toluolica* Tol2 [29]. To the best of our knowledge, this is the first active monomeric PLP-dependent enzyme among the known fold type I and IV transaminases. The new monomeric transaminase (*Dtol*TA) is stereoselective and active toward D-alanine and (*R*)-phenylethylamine. We have determined the crystal structure of the enzyme and studied its monomeric organization in detail. The results show that the active site of the transaminase, formed by a single subunit, is active in PLP-catalyzed deamination but is insufficient for double-substrate transamination. This study was undertaken to analyze the catalytic properties of transaminase in the monomeric form and to underscore the importance of the enzyme’s dimeric organization in transaminase catalysis.

## 2. Results and Discussion

### 2.1. Enzyme Identification, Expression, and Purification

Searching for new transaminases of PLP fold type IV revealed three sequences in the complete genome of *D. toluolica*: BCAT (GenBank ID: WP_014956217.1), aminodeoxychorismate synthase component I (WP_014958710.0), and putative transaminase (WP_014959278.1). The sequence of the transaminase, named *Dtol*TA, contained a specificity-determining sequence motif [30,31], which differed from the sequence motifs of the known transaminases of PLP fold type IV (Table S1). This motive was not observed in the known PLP-dependent isochorismate lyases of fold type IV as well.

**Figure 2:**
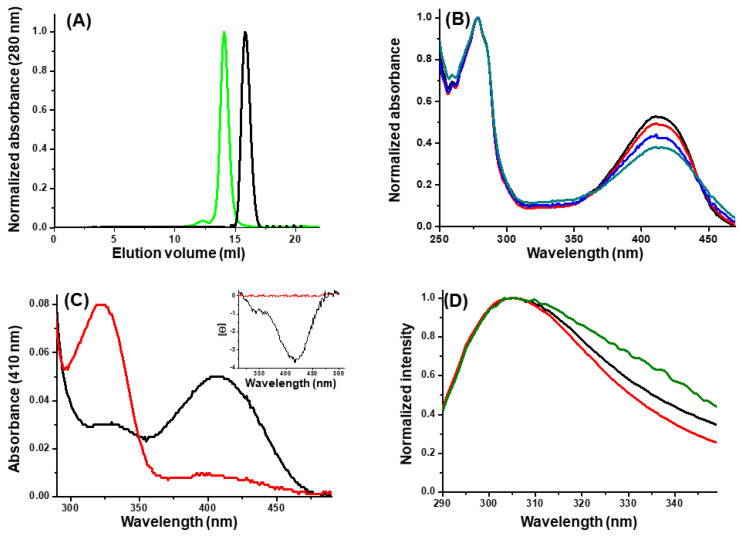
Properties of *Dtol*TA. (**A**) Gel filtration of *Dtol*TA (black, monomer) and, for comparison, of TA from *Haliscomenobacter hydrossis* (green, dimer) [32]; (**B**) spectra of holoenzyme *Dtol*TA at pH 5.5 (black), 6.5 (red), 7.5 (blue), and 8.5 (green); (**C**) UV-Vis spectra of 15 µM *Dtol*TA in holo form and after incubation with 1 mM D-alanine, Inset: CD spectra of 30 µM holoenzyme and after incubation with 1 mM D-alanine; (**D**) fluorescence emission spectra (excitation at 280 nm) of holoenzyme (black), apoenzyme (red), and apoenzyme incubated with 100 µM PLP (green).

A transaminase *Dtol*TA was cloned and expressed in a soluble form in *E. coli* and purified to homogeneity (Figure S1). The molecular weight of the purified *Dtol*TA determined by gel filtration was approximately 34 kDa, indicating *Dtol*TA as a monomer in solution (Figure 2A). The absorption spectrum of the holo form of *Dtol*TA had two characteristic peaks: one at 278 nm and another at 411 nm (Figure 2B). The latter indicated the formation of a Schiff base with PLP. No spectral changes were observed between pH 5.5–8.5 (Figure 2B). The maximum at 411 nm referred to the protonated form of the internal aldimine over the entire pH range 5.5–8.5.

### 2.2. Enzyme Activity, Substrate Specificity, and Enantioselectivity

**Scheme 1:**
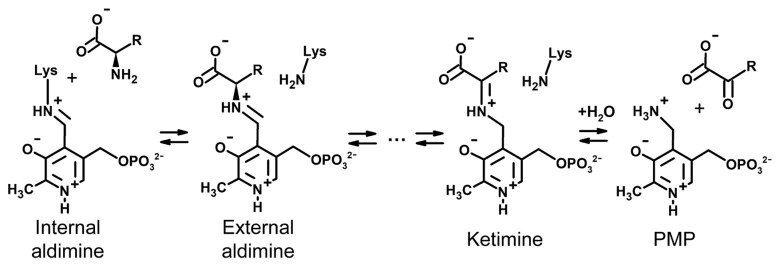
The transamination half-reaction with D-alanine.

No activity of *Dtol*TA was observed in the overall transamination reaction with the benchmark substrates of transaminases of PLP fold type IV: D-alanine, D-glutamate, L-leucine, and (*R*)-1-phenylethylamine (R-PEA) as amino donors and pyruvate and α-ketoglutarate as amino acceptors. No *Dtol*TA-catalyzed transamination was detected between D-alanine or R-PEA and various keto compounds, namely, 2-oxobutyrate, 4-methyl-2-oxovalerate, 3-methyl-2-oxovalerate, 2-pentanone, 2,3-butandione, isobutyraldehyde, and propanal. The substrate conversion came down to the consumption of only an (*R*)-stereospecific amino donor and PLP. In other words, only the first half-reaction (Scheme 1), namely, the deamination of D-amino acid or R-PEA accompanied by the transfer of the amino group to the cofactor with the formation of pyridoxamine-5′-phosphate, was observed in the reaction medium.

While *Dtol*TA was active in half-reactions with D-alanine, D-glutamate, and R-PEA in 50 mM Na phosphate buffer, supplemented with 50 mM NaCl, pH 8.5, at 30 °C, no half-reaction was observed with D-leucine, D-phenylalanine, D-lysine, D-homoserine, L-alanine, L-leucine, L-glutamate, (*S*)-phenylethylamine, etc., in the same conditions (Table S2). It should be noted that the calculated pI of the *Dtol*TA is 9.38 (the ExPASy World Wide Web server [33]). We tested the activity of the enzyme in 50 mM Na bicarbonate buffer, pH 10.0, at 30 °C. The specificity of *Dtol*TA was the same under these alkaline conditions, but the holoenzyme dissociation and free PLP accommodation (as seen from the holoenzyme spectra) increased significantly compared to at pH 8.0. The enzyme was active toward D-alanine at pH 4.5. However, rapid holoenzyme dissociation was observed as well.

We evaluated the activity of *Dto*lTA in half-reactions in the pH range 7.0–8.0. For D-alanine deamination by *Dtol*TA, the values of k_max_/K_D_^app^ were found to be 29 ± 1 M^−1^s^−1^ (pH 7.0) and 37 ± 1 M^−1^s^−1^ (pH 8.0) in 50 mM Na phosphate buffer, supplemented with 50 mM NaCl, at 30 °C. For R-PEA and D-glutamate deamination by *Dtol*TA, the values of k_max_/K_D_^app^ were 0.015 ± 0.005 M^−1^s^−1^ and 0.020 ± 0.001 M^−1^s^−1^, respectively. In 50 mM HEPES buffer, pH 8.0, supplemented with 50 mM NaCl, the k_max_/K_D_^app^ value for D-alanine deamination by *Dtol*TA was also 37 ± 1 M^−1^s^−1^ at 30 °C. Saturation was not achieved with the substrates studied: precipitation of the *Dtol*TA was observed at high substrate concentrations. Scanning of the half-reaction of *Dtol*TA with D-alanine showed the accumulation of free PMP in the solution (Figure 2C). Bound E-PMP was also not detected by the CD method (Figure 2C, insert). Apparently, the E-PMP form of *Dtol*TA was unstable and dissociated in the course of the half-reaction. The second half-reaction in the overall transamination reaction conditions cannot be achieved with free PMP. Thus, in monomeric form, the transaminase is stereospecific and capable of catalyzing a first half-reaction similar to dimeric transaminases. *Dtol*TA is inferior to dimeric transaminases in stabilizing the holoenzyme with the reduced cofactor—pyridoxamine 5′-phosphate—and the Michaelis complex of the holoenzyme and an amino acceptor. The dimeric organization appears to be crucial for the overall transamination reaction.

### 2.3. Holoenzyme and Apoenzyme Stability

The holoenzyme (E-PLP form) was stable in 50 mM Na phosphate buffer, supplemented with 50 mM NaCl, at pH 6.5, 7.0, and 8.0, at 30 °C. No changes in the holoenzyme spectra were observed in 120 min. In 50 mM Na acetate buffer, pH 4.5, and 50 mM Na bicarbonate buffer, pH 10.0, the holoenzyme dissociation was fast. The apoenzyme was folded (Figure 2D) but tended to aggregate. It was obtained by incubation of *Dtol*TA with the excess of D-alanine, and after desalting, a slow aggregation process was observed. The solutions of 10 mkM apoenzyme and lower concentrations were stable. Incubation with PLP led to the recovery of active holoenzyme (Figure 2D). From the titration of the apoenzyme by PLP, the data for quenching of intrinsic fluorescence versus PLP concentration fitted to Equation (3) yielded an apparent Kd value for the holoenzyme of 30 ± 4 µM. The protein globule of *Dtol*TA was stable; the midpoint temperature of thermal denaturation (T_0.5_) was 60.2 ± 0.5 °C. While NaCl at a concentration of 20–50 mM stabilized *Dtol*TA, the higher concentrations of NaCl decreased the rate of half-reaction. Overall, the stability of monomeric *Dtol*TA is compared to that of dimeric transaminases [16,19,32,34,35], underscoring the importance of the dimer for catalysis rather than the stability of the transaminase.

### 2.4. The Overall Structure of DtolTA

Two crystal structures of *Dtol*TA were obtained, one at 2.6 Å (low resolution, space group F4_1_32) and another at 1.8 Å (high resolution, space groups P2_1_2_1_2_1_). The asymmetric unit of both structures contained one copy of a protein. The RMSD for the Cα atoms between the subunits of these structures was rather high (0.6 Å) due to different contacts with their symmetry mates and in the regions formed by residues 56–64, 95–102, and 110–120 (Figure S2). As part of the subunit had no electron density in the high-resolution structure (see in Materials and Methods), the low-resolution structure was used to describe the overall structure, while the high-resolution structure was used to describe the active site of *Dtol*TA. The closest structural homolog of *Dtol*TA was the D-amino acid transaminase from *Bacillus* sp. *YM-1* (*bs*DATA) (Table 1). According to the crystallographic contacts, *Dtol*TA is a monomer, and no functional dimer similar to *bs*DATA was found in the crystal cell based on contact analysis.

**Figure 3:**
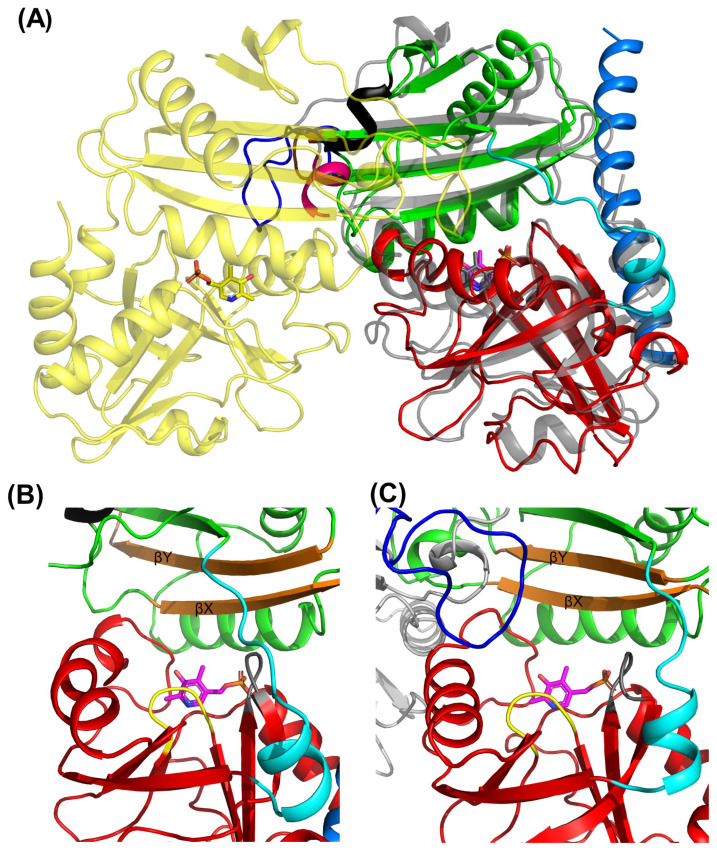
The overall structure of *Dtol*TA. (**A**) The superposition of the monomer of *Dtol*TA (colored) and the subunit of *bs*DATA (gray). The small domain of *Dtol*TA is shown in green, the large domain in red, the interdomain loop in cyan, the C-terminal α-helix in light blue, the O-pocket α-helix in black, and the O-pocket loop in brown. The PLP molecule is shown in magenta sticks. The O-pocket α-helix and loop of the subunit in *bs*DATA are shown in pink and blue, respectively. The adjacent subunit in the *bs*DATA dimer is shown in semitransparent yellow. (**B**) The active site of *Dtol*TA. The color scheme is similar to panel (**A**) with additional color representations: βX- and βY-strands are in orange and labeled, β-turn1 is yellow, and β-turn2 is gray. (**C**) The active site of *bs*DATA. The color scheme is similar to panel (**B**), the O-pocket loop of the adjacent subunit is shown in blue, and the adjacent subunit is shown in gray. Panels (**B**,**C**) are shown in the same orientation.

Similarly to the known transaminases of PLP fold type IV, the subunit of *Dtol*TA consists of two α/β domains (Figure 3A): a small domain (residues 1–107) and a large domain (residues 122–286) connected by the interdomain loop (residues 108–121). The positions of the secondary structural elements forming the active site, the βX-strand (33–40), βY-strand (87–96), β-turn1 (171–174), and β-turn2 (233–236), are similar to those of known transaminases of PLP fold type IV (Figure 3B,C). The *Dtol*TA monomer is distinguished by the O-pocket loop (residues 97–100), which is significantly shorter than that in *bs*DAAT (residues 92–110), the C-terminal α-helix (residues 256–286), which is approximately twice as large as that in *bs*DATA (residues 263–277), and, finally, by the unique position of the O-pocket α-helix (residues 22–27) (Figure 3A).

**Figure 4:**
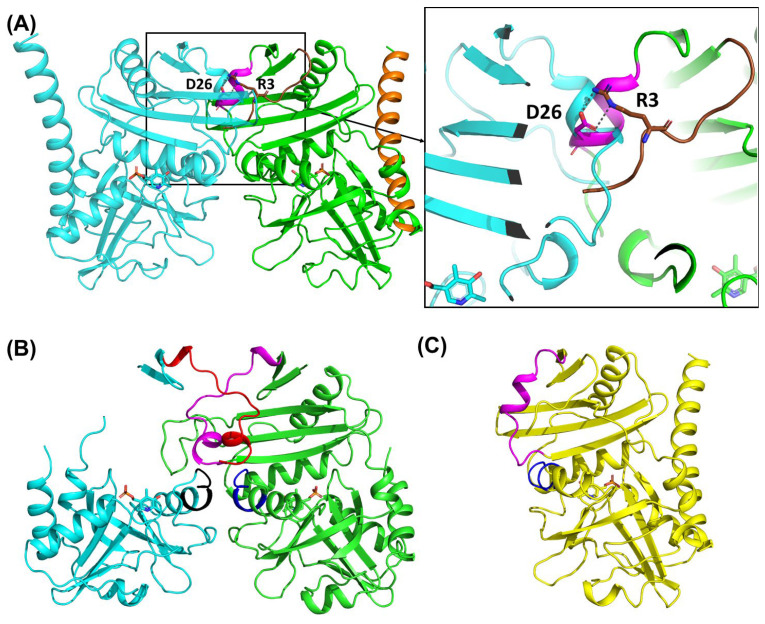
Comparison of classical functional dimers of PLP fold type IV transaminases and monomeric *Dtol*TA. (**A**) Modeling of the classical dimer for *Dtol*TA. The superposition of the *Dtol*TA monomers on the subunits of the *bs*DAAT dimer. The subunits are shown in different colors: the α-helix 22–27 is shown in magenta, the N-terminal loop in brown, and the C-terminal α-helix in orange. A salt bridge is indicated by gray dashed lines. Regions 1 and 2 of (**B**) *bs*DATA and (**C**) *Dtol*TA. Regions 1 and 2 of the green and cyan subunits in panel (**B**) are shown in magenta and blue and red and black, respectively. Regions 1 and 2 of *Dtol*TA in panel (**C**) are shown in magenta and blue, respectively. The green subunit in panel (**B**) and the yellow subunit in panel (**C**) are shown in the same orientation. Residues 30–117 of the cyan subunit are transparent for better viewing.

**Figure 5:**
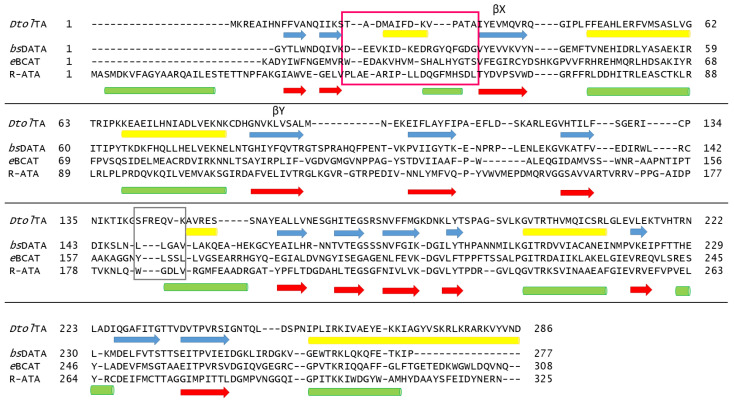
Structure-based sequence alignment of *Dtol*TA and homologous *bs*DATA, eBCAT, and R-ATA from *A. terrus* (Table 1). Secondary structure elements were assigned by PDBeFOLD [36] and are shown below the amino acid sequence as follows: for *Dtol*TA, β-strands and α-helices as blue arrows and yellow cylinders, respectively; for *bs*DATA, eBCAT, and R-ATA, red arrows and green cylinders, respectively. Regions 1 and 2 are shown in pink and gray rectangles, respectively.

To explain the monomeric form as the only possible one for *Dtol*TA, its ‘classical dimer’ was modeled by superimposing two *Dtol*TA monomers and both subunits of the *bs*DAAT functional dimer (Figure 4A). Structure-based sequence alignment of *Dtol*TA and homologous transaminases was also carried out (Table S2, Figure 5). Two regions (regions 1 and 2) important for dimerization in classical transaminases were identified and analyzed. In *Dtol*TA, region 1 (Figure 4B,C and pink rectangle in Figure 5) was shorter than in other transaminases and included an α-helix (residues 22–27), so that the residues of region 1 did not protrude from the subunits as in homologous transaminases (Figure 4 and Figure 5). In addition, in *Dtol*TA, the position of α-helix 22–27 was reinforced by the salt bridge Asp26–Arg3. This salt bridge also positioned the N-terminus directly at the dimer interface, preventing dimer formation. As for region 2 (gray rectangle in Figure 5), in *Dtol*TA (residues 139–146), it is a loop, while in the other transaminases, it is an α-helix, in which the amino acid residues interact with those from region 1 of the adjacent subunit (Table S3). Other structural elements involved in the formation of the dimer interface in *bs*DATA, *e*BCAT, and R-ATA have the same spatial arrangement as in *Dtol*TA. It is worth noting that the long C-terminal α-helix of *Dtol*TA does not affect the formation of the classical dimer, but appears to contribute to the stabilization of the monomer via hydrogen bonds between its residues and residues of the small and large domains: E265-R40, Y271-E48, and R275-nitrogen atom of the main chain of F47.

### 2.5. The Active Site of DtolTA

**Figure 6:**
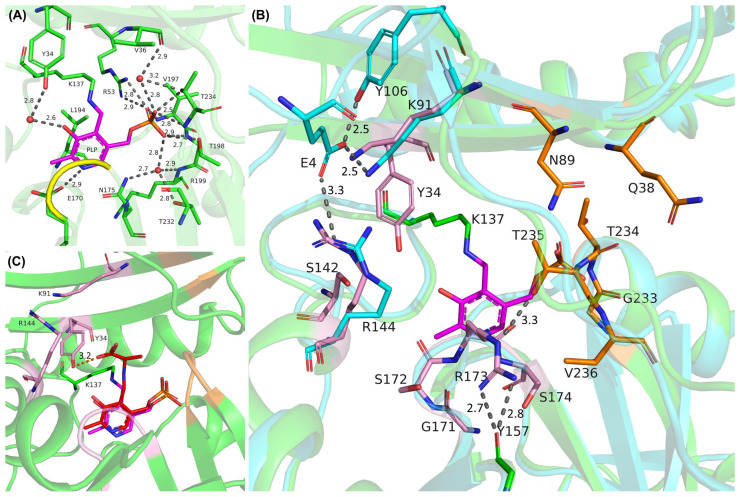
The active site of *Dtol*TA. (**A**) Binding of PLP molecule in the active site of *Dtol*TA. Water molecules are shown as red spheres. Hydrogen bonds are shown as dashed gray lines and correspondent distances are in angstroms. β-turn1 (R173 and S174) is colored in yellow. (**B**) Organization of the substrate-binding pockets. Superposition of the high-resolution (green, cartoon) and low-resolution (cyan, cartoon) structures. For the high-resolution structure, active site residues are shown in sticks and colored as follows: P-pocket residues in orange, O-pocket residues in pink, and PLP in magenta. For clarity, in the case of the low-resolution structure, only residues with conformations different from the high-resolution structure are shown (cyan, sticks). (**C**) Superposition of *Dtol*TA and *bs*DATA complexed with D-alanine (PDB ID 3DAA). For clarity, only the ligand of *bs*DATA is shown (shown as red sticks). The color scheme of *Dtol*TA is similar to that shown in panel (**B**). Distance between OH(Y34) and the oxygen atom of the α-carboxyl group of D-alanine is shown as a dashed orange line and labeled in angstroms.

The *Dtol*TA monomer has an active site formed by the residues of the small and large domains. The PLP molecule is clearly visible in the electron density (Figure S3) and is bound to K137 via a Schiff base linkage. The phosphate moiety of PLP is coordinated by hydrogen bonds with the side chains of R53, T198, and T234, backbone nitrogen atoms of V197, T198, and T234, and water-mediated hydrogen bonds with V36, N175, R199, and T232 (Figure 6A). Interestingly, the pyridine ring of PLP is sandwiched by the side chain of L194 and the backbone atoms of R173 and S174 (β-turn1, shown as yellow in Figure 6A). E170 forms a hydrogen bond with the N1 atom of the pyridine ring of PLP. Y34 coordinates the phenyl group of PLP via the water molecule. Overall, the binding of PLP in *Dtol*TA is typical for PLP fold type IV transaminases; the interaction of the positively charged pyridine N1 atom and E170 together with the ring-stacking interactions with the PLP pyridine ring increases the electron-withdrawing capacity of the cofactor, favoring the stabilization of the carboanionic intermediate in catalysis [2,37].

Regarding the O- and P-pocket active site organization in PLP fold type IV transaminases, residues Y34, K91, S142, R144, and β-turn1 (^171^GSRS^174^) form the O-pocket (Figure 6B). The residues E4 K91, Y106, and R144 are bound by a hydrogen bond network to build the left side of the O-pocket. Residues Q38, N89, and β-turn2 ^233^GTTV^236^ (Figure 6B) form the P-pocket in *Dtol*TA. The P-pocket lacks a positively charged group that could fix the γ-carboxylate group of the substrate α-ketoglutarate and D-glutamate similar to K35 in *bs*DATA. Superposition of *Dtol*TA and *bs*DATA complexed with D-alanine (PDB ID 3DAA, Figure 6C) showed that Y34 could coordinate the α-carboxylate group of D-alanine. The residue R173 might participate in the binding of the α-carboxylate group; however, in both *Dtol*TA structures the backbone oxygen of Y157 locks the side chain of R173 outside the active site, removing it from the pockets. Similarly, the residues K91 and R144 are anchored outside by the hydrogen bonds with the residue E4.

Overall, the monomeric *Dtol*TA should be considered as a transaminase because (1) it is of the fold type IV organization, and for these PLP-dependent enzymes, only transaminase or isochorismate lyase activity are shown; (2) the PLP protonation state and coordination is typical of transaminases (namely, the hydrogen bond between N1 and the carboxylate group of the glutamate residue, the coordination of the phenyl oxygen, and the pyridine ring stacking with the side chain of L194 and backbone atoms of β-turn1), and (3) the O-pocket of *Dtol*TA has arginine and tyrosine residues, which usually coordinate the α-carboxylate group of substrates in transaminases. The integrity of the monomer is supported by the interdomain loop, the extended C-terminal helix, and, apparently, the hydrogen bond network E4-K91-Y106-R144. It is noteworthy that the active site of *Dtol*TA is open, similar to the known dimeric DATA [13,32,35,38]. For other PLP fold type IV transaminases, BCATs, and R-ATAs, closure of the active site during catalytic turnover is suggested by the observed shift in position of the interdomain and O-pocket loops in crystal structures of complexes with substrates and the double positions of these loops in holoenzymes [17,25,28]. The closure of the active site is believed to protect the catalytic turnover from side reactions (e.g., racemization) and cofactor leakage [15,28,39]. Interestingly, no side reaction products were detected in DATAs with an open active site, but cofactor leakage was observed [38,40,41,42]. The inability of *Dtol*TA to catalyze the overall transamination reaction may be due to an unfavorable ratio between the rates of PMP leakage and keto acid association in the Michaelis complex formation step.

### 2.6. Analysis of the Variants R144I and K91A

**Figure 7:**
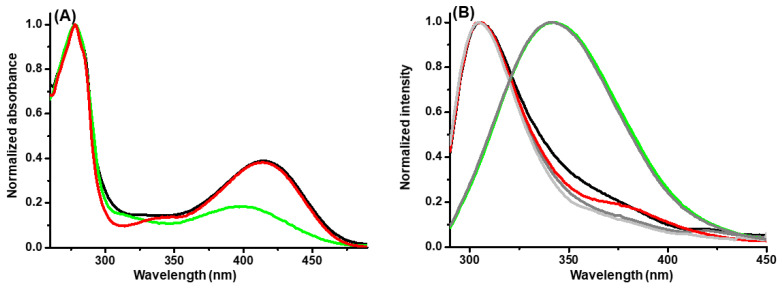
Absorbance (**A**) and fluorescence (**B**) spectra of holo forms of WT *Dtol*TA (black) and its variants R144I (red) and K91A (green). Fluorescence spectra of the apo forms of WT and K91A (dark gray) and R144I (light gray).

In the active site of *Dtol*TA, residues R144 and K91 are involved in the hydrogen bond network (Figure 6A) and seem to be responsible for the integrity of the O-pocket and the whole active site and for binding of the substrate α-carboxylate group. We investigated the effects of the R144I and K91A substitutions on the activity of *Dtol*TA in the half-reaction with D-alanine and on the stability of the holoenzyme. The R144I variant was stable (T0.5 was 60.5 ± 0.1 °C), and the UV-Vis and fluorescence spectra of the holo form were similar to those of the WT (Figure 7). The k_max_/K_D_^app^ of the half-reaction of R144I with D-alanine and the apparent dissociation constant Kd(PLP) for the R144I holoenzyme were similar to those of WT (29 ± 1 M^−1^s^−1^and 43 ± 2 µM, correspondingly). The K91A substitution caused a decrease in both the activity and stability of the holoenzyme (Figure 7). The latter appears to be a mixture of apoenzyme and free PLP in phosphate buffer, pH 8.0 (Figure 7A). In other words, the K91A substitution destroyed the cofactor-binding ability in *Dtol*TA. The variant apoenzyme was stable and able to convert D-alanine in the presence of PLP (Figure S4). The apparent values of k_max_/K_D_^app^ for D-alanine deamination by the variant K91A were estimated to be 2.4 ± 0.7 M^−1^s^−1^ in 50 mM Na phosphate buffer, pH 8.0, supplemented with 50 mM NaCl, at 30 °C. The red shift in the fluorescence spectrum of K91A indicated the increase in hydrophilicity of the tyrosine environment (Figure 7B) and, apparently, both Y106 and Y34 became more accessible to the solvent compared to WT *Dtol*TA (Figure S5). The observed rearrangement in the active site appeared to affect the stability of the Schiff bond between the PLP and the catalytic lysine. Overall, the hydrogen bond network E4-K91-Y106-R144 (Figure S6A) is important not only for the integrity of the O-pocket but also for the proper binding of PLP.

## 3. Material and Methods

### 3.1. Cloning, Expression, and Purification of the Recombinant DtolTA

The PLP type IV transaminase encoded by the TOL2_C39420 gene (286 a.a., 32.4 kDa) was identified in the complete genome sequence of *D. toluolica* (strain DSM 7467/Tol2) [29]. The optimized sequence (http://genomes.urv.es/OPTIMIZER/, accessed on 1 February 2023) was synthesized with 5′ and 3′ restriction sites NdeI and HindIII by ATG Service Gene (Saint Petersburg, Russia) and then cloned into the pET-21d vector (Novagen, Darmstadt, Germany), modified as described in [43], to create a fusion protein with a (His)_6_-tag and a TEV protease site at the N-terminal. The *E. coli* Rosetta (DE3) pLysS cells (Novagen, Germany) with the expression vector pET-21d_ DtolTA were grown in LB medium containing 34 μg/mL chloramphenicol and 100 μg/mL ampicillin (Panreac-AppliChem, Darmstadt, Germany) at 37 °C until obtaining an OD_600_ of 0.8. The expression of protein was started by 0.2 mM IPTG and continued for one hour at 24 °C.

The cells were collected by centrifugation, resuspended in a 50 mM Tris_HCl buffer, pH 8.0, supplemented with 500 mM NaCl, 2 M urea, 20 mM imidazole, 5 mM β-mercaptoethanol, 10% (*v*/*v*) glycerol, 0.2 μg/mL lysozyme, 100 μM PLP, and 1 mM PMSF, and disrupted by sonication. The crude cell extract was treated with 0.1 mg DNAse (Sigma-Aldrich, St Louis, MO, USA), centrifuged for 45 min at 18.500× *g* at 4 °C, and the supernatant was filtered through a 0.45 μm filter (Millipore, Burlington, MA, USA), and then was loaded onto a 5 mL HisTrap HP column (Cytiva, Marlborough, MA, USA) equilibrated with 50 mM Tris-HCl buffer, pH 8.0, containing 500 mM NaCl, 20 mM imidazole, and 0.1% (*v*/*v*) Triton X-100. Recombinant (His)_6_-tagged *Dtol*TA was eluted by a linear gradient of imidazole (20 to 500 mM) in the 50 mM Tris-HCl buffer, pH 8.0, containing 500 mM NaCl. The target *Dtol*TA was transferred to storage buffer (50 mM Tris_HCl buffer, pH 8.0, containing 100 mM NaCl and 100 μM PLP). The DtolTA stored at −20 °C with the 50% glycerol.

For biochemical characterization, (His)_6_-tagged *Dtol*TA was used. The variants of *Dtol*TA were created by single-primer site-directed mutagenesis, as described in [32]. Oligonucleotides listed in Table S4 were used as primers for mutagenesis. The variants were produced and purified as described for WT *Dtol*TA. The amino acid sequences were checked by MALDI-TOF MS analysis (UltraFlextreme Bruker Daltonik, Bremen, Germany).

For crystallization, the (His)_6_-tag was removed using (His)_6_-tagged TEV protease. Recombinant (His)_6_-tagged *Dtol*TA was mixed with (His)_6_-tagged TEV protease (1 mg per 10 mg of the transaminase) and incubated overnight at 4 °C in 50 mM Tris-HCl buffer, pH 7.5, supplemented with 10% (*v*/*v*) glycerol, 1 mM EDTA, 5 mM β-mercaptoethanol, and 100 μM PLP. Next, the reaction mixture was dialyzed against 50 mM Tris-HCl buffer, pH 7.5, supplemented with 500 mM NaCl, 20 mM imidazole, 5 mM β-mercaptoethanol, and 20 μM PLP and applied to a HisTrap HP column (Cytiva, USA). The *Dtol*TA without (His)_6_-tag was collected in the flow-through mode, transferred into 20 mM HEPES buffer, pH 7.5, containing 50 mM NaCl, concentrated, and applied to a Source 30S column (Cytiva, USA), equilibrated with the same buffer, and eluted with a linear NaCl gradient (50–500 mM). The collected fraction was concentrated and finally purified using gel filtration on a Superdex 200 10/300 GL column (Cytiva, USA) equilibrated in crystallization buffer (50 mM HEPES buffer, pH 7.5, supplemented with 100 mM NaCl, 100 μM PLP, and 1 mM DTT). The resulting fraction of recombinant *Dtol*TA was concentrated up to 15 mg/mL and frozen at −70 °C.

The homogeneity of *Dtol*TA was analyzed by SDS-PAGE (12%). The concentration was determined spectrophotometrically at 280 nm using a calculate extinction coefficient (https://web.expasy.org/protparam/, accessed on 1 September 2024).

### 3.2. Half-Reaction Assay

The PLP forms of *Dtol*TA and the R144I variant were obtained by incubating it with an excess of PLP for 1 h at 25 °C. The mixture was then transferred into 50 mM K-phosphate buffer, pH 8.0, supplemented with 50 mM NaCl, using a 5 mL desalting column (Cytiva, USA). Half-reactions between the PLP form of *Dtol*TA (30–35 μM) and D-alanine (0–100 mM), D-glutamate (0–100 mM), and R-PEA (0–100 mM) were monitored spectrophotometrically at 411 nm by measuring the decrease in aldimine concentration in 50 mM Na phosphate buffer, pH 8.0, supplemented with 50 mM NaCl at 30 °C using an Evolution 300 UV-Vis spectrophotometer (Thermo Scientific, Waltham, MA, USA). The rate constants of half-reactions were determined by fitting Equation (1):

(1) $$A_{t}=A_{\infty }+∆Aexp(-k_{obs}t)$$

where $A_{t}$ is the absorbance at time *t*, ∆A is the difference between the absorbance at *t* = 0 and *t* = ∞, $A_{\infty }$ is the final absorbance, and k_obs_ is the observed rate constant.

The specificity constants k_max_/K_D_^app^ were obtained from the dependences of the k_obs_ on the substrate concentration that are described by Equation (2):

(2) $$k_{obs}=\frac{k_{max}\left[S\right]}{K_{D}^{app}+\left[S\right]}$$

All measurements were performed at least in triplicate, and the data were analyzed using Origin 8.0 software (OriginLab, Northampton, MS, USA).

### 3.3. Enzyme Activity Assay

The activity of *Dtol*TA toward D-alanine and D-glutamate in transamination reactions was measured spectrophotometrically using the second enzymatic reaction with lactate dehydrogenase from rabbit muscle (Roche Diagnostic GmbH, Mannheim, Germany) or recombinant (*R*)-2-hydroxyglutarate dehydrogenase from *Acidaminococcus fermenats*, respectively, in the presence of NADH [32,38]. The decrease in absorbance at 340 nm (ε(NADH) = 6.22 mM^−1^ cm^−1^) was measured using an Evolution 300 UV-Vis spectrophotometer to monitor the reaction’s progress. The activity of *Dtol*TA with (*R*)-PEA as the amino donor was measured by a direct photometric assay by detecting acetophenone production (acetophenone-assay) at 245 nm (ε(Acetophenone) = 11.6 mM^−1^ cm^−1^) in a cuvette with 0.5 cm optical path length, using Evolution 300 UV-Vis spectrophotometer.

### 3.4. Determination of the Dissociation Constant of the PLP Form of the Holoenzyme

The binding of PLP to the *Dtol*TA WT and the R144I variant was followed by monitoring changes in the intrinsic tyrosine fluorescence (no tryptophan residues are in the amino acid sequence). The apparent dissociation constants of the PLP form of holoenzyme for the *Dtol*TA WT and the R144I variant were determined by measuring the quenching of the tyrosine fluorescence (excitation at 280 nm and emission at 305 nm) following the incubation of the apo form of the variants (4–10 μM) with PLP at concentrations ranging from 4 to 200 μM for one hour at 25 °C in 50 mM K-phosphate buffer, pH 8.0, supplemented with 50 mM NaCl. Tyrosine fluorescence was acquired using a spectrofluorometer FluoroMax-4 (Horiba Scientific, Kyoto, Japan). The PLP dissociation constant (K_d(PLP)_) was determined by fitting the data to Equation (4):

(3) $$Y=Y_{max}\times \frac{\left[E\right]+\left[PLP\right]+K_{d\left(PLP\right)}-\sqrt{{\left(\left[E\right]+\left[PLP\right]+K_{d\left(PLP\right)}\right)}^{2}-4[E][PLP]}}{2[E]},$$

where [E] and [PLP] represent the total concentrations of the apo form and PLP, respectively, Y is the tyrosine fluorescence quenching at the PLP concentration [PLP], and Y_max_ is the tyrosine fluorescence quenching when all enzyme molecules are complexed with PLP. All measurements were performed at least in triplicate, and the data were analyzed using the Origin 8.0 software.

### 3.5. Analysis of Thermal Stability of the DtolTA

Circular dichroism spectroscopy was used to examine the thermal unfolding. Molar ellipticity [θ] changes at 220 nm were acquired for 10 μM *Dtol*TA in 20 mM Na phosphate buffer, pH 8.0, containing 20 mM NaCl and 30 µM PLP in a 1 mm optical path cuvette using a Chirascan instrument, equipped with a Peltier thermostatic cell holder (Applied Photophysics, Surrey, UK). Heating from 20 to 80 °C was carried out at a rate of 1 °C/min. The midpoint temperature of thermal denaturation (T_0.5_) was calculated using Boltzmann’s equation. The data were analyzed using the Origin 8.0 software.

### 3.6. Crystallization and Data Collection

Primary crystallization screening was carried out by the “hanging drop” vapor diffusion method on a robotic system (Rigaku Americas Corporation, The Woodlands, TX USA) using 96-well VDX plates (Hampton Research, Aliso Viejo, CA, USA) and commercial crystallization screens from Hampton Research (Aliso Viejo, CA, USA) and Molecular Dimensions Inc (Holland, OH, USA). The *Dtol*TA (15 mg/mL) in crystallization buffer (see in 3.1) was mixed with the crystallization reagents in the ratios 1:1 (0.2 μL drop volume) and 2:1 and 1:2 (0.3 μL drop volume). The reservoir contained 50 µL of the precipitant solution. The initial crystallization hit was observed under the following conditions: 0.1 M Bis-tris propane, pH 6.5, 200 mM sodium nitrate, and 20% PEG 3350 at a 1:1 ratio (*Dtol*TA (low resolution)) and 0.1 M Bis-tris, pH 5.5, and 0.1 M ammonium acetate 17% PEG 10,000 at a 1:1 ratio (*Dtol*TA (high resolution)) at 277 K. Further optimization of crystal growth was achieved using the “hanging drop” vapor diffusion method in 24-well VDX plates (Hampton Research) with 500 μL of precipitant solution and a drop volume of 3 μL. Before diffraction, data collection crystals were soaked in a mother liquor containing 20% glycerol and immediately flash-frozen in liquid nitrogen. Datasets were collected at BL41XU beamline (Spring8, Japan) and ID23-1 beamline (ESRF, France) [44] for *Dtol*TA (low resolution) and *Dtol* (high resolution), respectively, at 100 K. The data processing for *Dtol*TA (low resolution) was performed using the Dials program from the CCP4 package v.7.1.013 [45], while for the *Dtol*TA (high resolution), the XDS package Version 5 February 2021, BUILT=20210323 [46] was used. Space groups were suggested as F4_1_32 and P2_1_2_1_2_1_ for *Dtol*TA (low resolution) and *Dtol*TA (high resolution), respectively (Table 2), by Pointless [47].

### 3.7. Structure Solution and Refinement

The *Dtol*TA (low resolution) structure was solved using the MOLREP program [48] by the molecular replacement method and the corresponding model prepared with AlphaFOLD2 [49], while the *Dtol*TA (high resolution) structure was solved using the *Dtol*TA (low resolution) structure as the initial model. One copy of the protein was observed in an asymmetric unit of both structures. A total of 30 residues at the N-terminus and 9 residues at the C-terminus had no electron density in *Dtol*TA (high resolution), while in *Dtol*TA (low resolution), the entire polypeptide chain was clearly visible.

The refinement of both structures was carried out using the REFMAC5 program of the CCP4 suite [45]. The manual rebuilding of the models and the visual inspection of electron density maps were carried out using the COOT interactive graphics program [50]. The hydrogen atoms in fixed positions and the isotropic B-factor were used during the refinement. For *Dtol*TA (low resolution) TLS was introduced during the final refinement cycles. The protein subunits of both structures have a similar fold (corresponding pairwise RMSD not exceeding 0.55 Å).

### 3.8. Structure Analysis and Validation

The COOT program and the PyMOL Molecular Graphics System, Version 4.6 (Schrödinger, New York, NY, USA), were used for the visual inspection of the modeled structures. The structure comparison and superposition were made using the PDBeFOLD program [36]. Finally, we constructed the dimer of *Dtol*TA using AlphaFold3 [51] (Figure S6). Unfortunately, the constructed dimer did not correspond to the classical functional dimer of TAs of PLP fold type IV.

## 4. Conclusions

The gene encoding the pyridoxal-5′-phosphate (PLP)-dependent transaminase of fold type IV has been identified in the complete genome sequence of the marine, aromatic-compound-degrading, sulfate-reducing bacterium *Desulfobacula toluolica* Tol2. The recombinant form of the transaminase, named *Dtol*TA, was obtained by heterologous expression in *E. coli* and studied by structural, kinetic, and spectral methods. Gel filtration and structural analysis revealed the monomeric organization of *Dtol*TA. To the best of our knowledge, this is the first monomeric transaminase among PLP fold type I and IV transaminases. The organization of the active site and the PLP binding mode were typical of PLP fold type IV transaminases, except for the arrangement of the O-pocket, which is formed by residues of two subunits in the functional dimers of PLP fold type I and IV transaminases. *Dtol*TA was (*R*)-stereoselective and deaminated D-alanine and (*R*)-phenylethylamine in half-reactions. However, the enzyme was unable to catalyze the overall transamination reaction between D-alanine and a α-keto acid. Monomeric *Dtol*TA was found to be inferior to dimeric DATA in stabilizing both the second holo form of transaminase with the reduced cofactor—pyridoxamine 5′-phosphate—which is the key point for the second half-reaction with α-keto acids, and the Michaelis complex between this holoenzyme and amino acceptor (α-keto acid or ketone). While the search for amino acceptors among α-keto acids was validated by the ability of *Dtol*TA to bind and deaminate D-amino acids, it may be that we failed to find the proper amino acceptor. Moreover, the openness of the active site of *Dtol*TA encourages further search for the amino acceptor. Anyway, we ascribed the transaminase function to *Dtol*TA based on (1) the structural similarity to the transaminases of PLP fold type IV, (2) the PLP protonation state and PLP coordination, and (3) the nature of the half-reaction as a transfer of the amino group from the amino acid or primary amine to the cofactor with keto acid or ketone production. Whether deamination activity is the only function of *Dtol*TA remains an open question.

It is noteworthy that no other transaminase of D-amino acids was observed in the *D. toluolica* genome. Deamination of D-amino acids and (*R*)-aromatic compounds with release of the amino group in the form of pyridoxamine 5′-phosphate (PMP) and accumulation of keto acids and ketones could be a possible function of *Dtol*TA in the cell, considering the ability of an anaerobic bacterium *D. toluolica* to degrade various aromatic compounds.

## Figures and Tables

**Table 1:** Superposition of the monomer of *Dtol*TA with the subunits of homologous transaminases of PLP fold type IV.

Transaminase from (Abbreviation, PDB ID)	Aligned Residues, %	RMSD Å	Sequence Identity, %
DATA from *Bacillus* sp. *YM-1 * (*bs*DATA, 4DAA)	86	2.1	25
BCAT from *E. coli* (*e*BCAT, 1I1K)	83	2.4	22
R-ATA from *Aspergillus terreus* (R-ATA, 4CE5)	86	2.4	19

**Table 2:** Data collection, processing, and refinement.

	*Dtol*TA (Low Resolution)	*Dtol*TA (High Resolution)
Diffraction source	BL41XU, Spring8	ID23-1, ESRF
Wavelength (Å)	0.90	0.88
Temperature (K)	100	
Detector	EIGER	PILATUS 6 M
Crystal-to-detector distance (mm)	250	250
Rotation range per image (°)	0.5	0.1
Total rotation range (°)	180	100
Space group	F4_1_32	P2_1_2_1_2_1_
*a*, *b*, *c* (Å)	250.09, 250.09, 250.09	37.85, 56.23, 118.63
α, β, γ (°)	90.00, 90.00, 90.00	90.00, 90.00, 90.00
Average mosaicity (°)	0	0.1
Resolution range (Å)	144.39–2.58 (2.69–2.58)	40.81–1.79 (1.83–1.79)
Completeness (%)	100.0 (100.0)	98.8 (96.2)
Average redundancy	66.9 (37.6)	3.6 (3.4)
〈*I*/σ(*I*)〉	17.3 (0.9)	13.4 (1.5)
Rmeas (%)	19.2 (917.4)	5.6 (78.8)
CC_1/2_	99.8 (44.6)	98.8 (96.2)
*R_fact_* (%)	25.1	19.3
*R*_free_. (%)	29.2	23.7
RMSD bonds (Å)	0.02	0.02
RMSD angles (°)	3.47	2.11
Ramachandran favored (%)	89.5	97.5
Ramachandran allowed (%)	8.1	2.0
PDB ID	9J0U	9J0V

## Data Availability

The data presented in this study are available on request from the corresponding author.

## Supplementary Materials

The following supporting information can be downloaded at: https://www.mdpi.com/article/10.3390/biom14121591/s1, Table S1: The specificity-determining sequence motifs of the canonical DAATs, BCATs, R-TAs, according to Höhne et al. [30], transaminase with expanded substrate specificity from *C. pusillum* and transaminase from *D. toluolica* (*Dtol*TA); Table S2: List of compounds analyzed as amino donors in half-reaction assay; Table S3: Amino acid residues involved in the dimer interface in bsDATA, eBCAT and R-ATA (polar contacts); Table S4: Sequences of primers used for mutagenesis, the mutations are underlined; Figure S1: SDS-PAGE of fractions of *Dtol*TA when expressed in *E. coli* and purified; Figure S2: Superposition of *Dtol*TA (low resolution, green) and *Dtol*TA (high resolution, magenta); Figure S3: The omit map (Fo-Fc, grey mesh at 3σ level) of PLP covalently bound with K137 in (A) *Dtol*TA (high resolution) and (B) *Dtol*TA (low resolution). PLP is colored in magenta; Figure S4: Half-reaction between the K91A variant (12.5 µM), 20 µM PLP and 10 mM D-alanine, spectrum of the apoenzyme is colored in black, the PLP decrease is observed at 390–400 nm, the PMP production at 324 nm; Figure S5: Structures and surfaces of the O-pocket region in (A) WT *Dtol*TA, (B) *Dtol*TA without sidechains of K91 (substitution K91A) and E4 and (C) *Dtol*TA without side chain of R144 (substitution R144I); Figure S6: (A,B) Dimers of *Dtol*TA (low resolution) predicted by AlphaFold3. (C) Functional dimer of bsDATA (PDB ID: 1DAA). Green subunits on all panels are shown in the same orientation.
