# Supplementary material for: From Structure to Function: Analysis of the First Monomeric Pyridoxal-5′-Phosphate-Dependent Transaminase from the Bacterium Desulfobacula toluolica
Source: Biomolecules. 2024 Dec 12;14(12):1591. doi: 10.3390/biom14121591 (PMC11673721; doi:10.3390/biom14121591)
Supplement: Supplementary file 1 [file biomolecules-14-01591-s001.zip › biomolecules-3328127-supplementary.pdf]

# Supplementary Materials

Tables S1-S4……………………………………………………………P.2-4
Figures S1-S5………………………………………………………….P.5-8

**Table S1.** The specificity-determining sequence motifs of the canonical DAATs, BCATs, R-TAs, according to Höhne et al. [1], transaminase with expanded substrate specificity from *C. pusillum* and transaminase from *D. toluolica* (*Dtol*TA).

| TA, (numbering corresponds to) | Specificity determining sequence motifs | |
| --- | --- | --- |
| | Motif 1 | Motif 2 |
| DAAT, (DAAT from *Bacillus* sp. YM-1) | $^{26}$FxxxxYxV[IVA][KR]$^{35}$ | $^{86}$HxY$^{88}$…$^{98}$[RK]xH$^{100}$ |
| BCAT, (BCAT from *Escherichia coli)* | $^{31}$YxxxxF[ED]Gx[KR]$^{40}$ | $^{95}$YxR$^{97}$…$^{107}$[LMVI]G[VL]$^{109}$ |
| R-ATA, (R-ATA from *Aspergillus terrus)* | $^{55}$HxxxxYD[VT]x[STAHP]$^{64}$ | $^{115}$[FY]V[EQAWNS]$^{117}$… $^{128}$[RKFGP]x[STANER]$^{130}$ |
| TA from *C. pusillum* | $^{51}$RxxxxFETIA$^{60}$ | $^{115}$GxK$^{117}$…$^{123}$GIEGEGR$^{129}$ |
| *Dtol*TA | $^{29}$PxxxxYEVRQ$^{38}$ | $^{89}$NVK$^{91}$… |

**Table S2. List of compounds analyzed as amino donors in half-reaction assay.**

| Compound | Structure | Activity (+/-) |
|---|---|---|
| D-alanine | 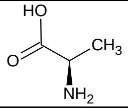 | + |
| L-alanine | 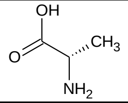 | - |
| D-leucine | 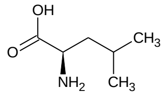 | - |
| D-glutamate | 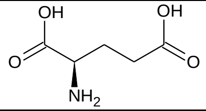 | + |
| D,L-phenylalanine | 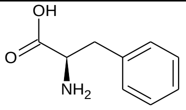 | - |
| D,L-ornithine | 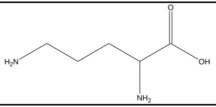 | - |
| D-homoserine | 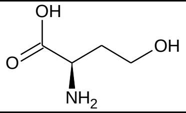 | - |
| D-lysine | 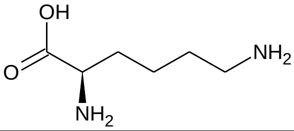 | - |
| β-alanine | 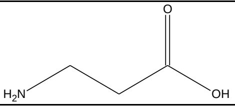 | - |
| (*R*)-phenylethylamine | 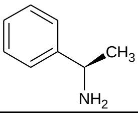 | + |
| (*R*,*S*)-1-methyl-3-phenylpropylamine | 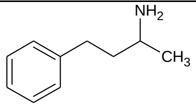 | - |
| (*R*)-ethylbenzylamine | 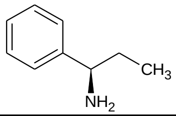 | - |
| (*R*)-1,2,3,4-tetrahydro-1-naphtalene | 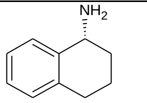 | - |
| Isobutylamine | 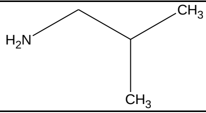 | - |
| (*R*,*S*)-2-amino-5-methylhexane | 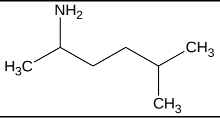 | - |
| (*R*,*S*)-2-amino-1-propanol | 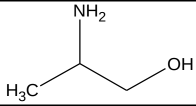 | - |

**Table S3.** Amino acid residues involved in the dimer interface in *bs*DATA, *e*BCAT and R-ATA (polar contacts). Residues from region 1 are colored in pink, from region 2 – grey.

| bsDATA | | eBCAT | | R-ATA | |
|---|---|---|---|---|---|
| Residue [atom] from subunit 1 | Residue [atom] from subunit 2 | Residue [atom] from subunit 1 | Residue [atom] from subunit 2 | Residue [atom] from subunit 1 | Residue [atom] from subunit 2 |
| ILE 17 [N] | ILE 17 [O] | HIS 23 [ND1] | ALA 20 [O] | ILE 46 [N] | ILE 46 [O] |
| TYR 114 [OH] | GLU 20 [OE2] | VAL 24 [N] | ALA 20 [O] | LEU 48 [N] | ALA 44 [O] |
| TYR 24 [OH] | ASP 21 [OD2] | VAL 22 [N] | VAL 22 [O] | SER 56 [N] | LEU 182 [O] |
| GLN 90 [NE2] | TYR 24 [O] | SER 34 [OG] | LEU 29 [O] | ARG 88 [NH1] | ASP 196 [OD1] |
| TYR 114 [OH ] | GLN 25 [OE1] | ARG 97 [NH2] | TYR 31 [OH] | ARG 88 [NH2] | ASP 196 [OD2] |
| LEU 149 [N] | PHE 26 [O] | ARG 175 [NE] | ASN 110 [OD1] | ARG 128 [NH1] | TRP 148 [O] |
| LEU 150 [N] | PHE 26 [O] | ASN 152 [ND2] | SER 145 [O] | ARG 168 [N] | GLY 173 [O] |
| LYS 156 [NZ] | GLN 101 [OE1] | ASN 152 [ND2] | ASN 147 [OD1] | TRP 184 [N] | HIS 55 [O] |
| LEU 140 [N] | ILE 137 [O] | ASN 147 [N] | ASN 152 [O] | TRP 184 [N] | GLN 183 [OE1] |
| GLY 27 [N] | LEU 147 [N] | GLY 32 [N] | GLY 161 [O] | GLY 185 [N] | GLN 183 [OE1] |
| LEU 149 [N] | ASN 148[ OD1] | ALA 20 [O] | VAL 24 [N] | ARG 189 [NH1] | PRO 172 [O] |
| LEU 150 [N] | ASN 148[ OD1] | VAL 22 [O] | VAL 22 [N] | ARG 189 [NH2] | GLY 173[O] |
| ARG 59 [NH2] | GLU 161 [OE1] | LEU 29 [O] | SER 34 [OG] | ALA 44 [O] | LEU 48 [N] |
| ARG 59 [NH2] | GLU 161 [OE2] | TYR 31 [OH] | ARG 97 [NH2] | ILE 46 [O] | ILE 46 [N] |
| ILE 17 [O] | ILE 17 [N] | SER 145 [O] | ASN 152 [ND2] | HIS 55 [O] | TRP 184 [N] |
| GLU 20 [OE2] | TYR 114 [OH] | ASN 147 [OD1] | ASN 152 [ND2] | TRP 148 [O] | ARG 128 [NH1] |
| ASP 21 [OD2] | TYR 24 [OH] | ASN 152 [O] | ASN 147 [N] | PRO 172 [O] | ARG 189 [NH1] |
| TYR 24 [O] | GLN 90[ NE2] | GLY 161 [O] | GLY 32 [N] | GLY 173 [O] | ARG 168 [N] |
| GLN 25 [OE1] | TYR 114 [OH] | | | LEU 182 [O] | SER 56 [N] |
| PHE 26 [O] | LEU 149 [N] | | | GLN 183 [OE1] | TRP 184 [N] |
| PHE 26 [O] | LEU 150 [N] | | | GLN 183 [OE1] | GLY 185 [N] |
| GLU 104 [OE2] | HIS 160 [NE2] | | | ASP 196 [OD1] | ARG 88 [NH1] |
| ILE 137 [O] | LEU 140 [N] | | | ASP 196 [OD2] | ARG 88 [NH2] |
| LEU 147 [O] | GLY 27 [N] | | | ILE 46 [N] | ILE 46 [O] |
| ASN 148 [OD1] | LEU 149 [N] | | | LEU 48 [N] | ALA 44 [O] |
| ASN 148 [OD1] | LEU 150 [N] | | | | |
| GLU 161 [OE1] | ARG 59 [NH1] | | | | |
| GLU 161 [OE2] | ARG 59 [NH2] | | | | |

**Table S4**. Sequences of primers used for mutagenesis, the mutations are underlined.

K91A.F; 5’–TCATGGTAATGTTGCCCTCGTGTCTGCTC–3’

CheckK91A.F; 5’–TGATCATGGTAATGTTGCC–3’

R144I.F; 5’–CAAAGGCAGCTTCATAGAACAGGTCAAGGCTG–3’

CheckR144I.R; 5’– CAGCCTTGACCTGTTCTA –3’

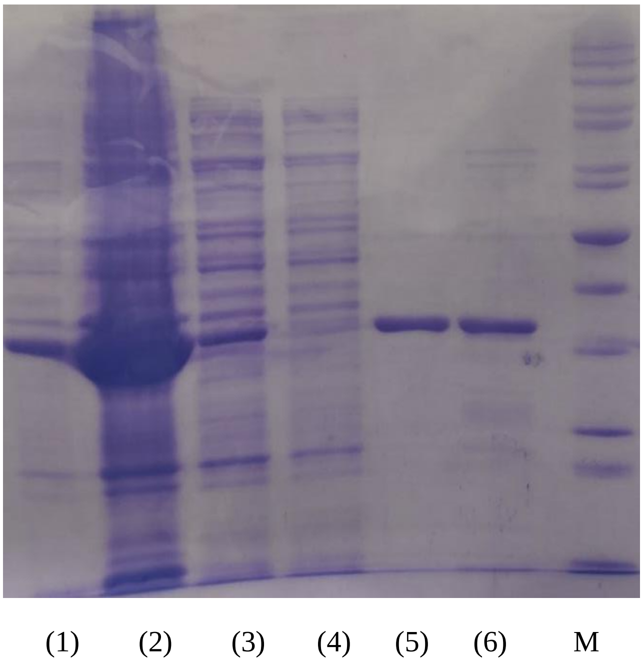


**Figure S1**. SDS-PAGE of fractions of *Dtol*TA when expressed in *E. coli* and purified. (1) cells after IPTG induction (2) lysate debris; (3) cells lysate after sonication and centrifugation; (4) flow-through after HisTrap HP column; (5) and (6) fractions of *Dtol*TA-His6TEVtag after HisTrap HP column; (M) Page Ruler Unstained Protein Ladder (Thermo Fisher Scientific, Waltham, MA, USA).

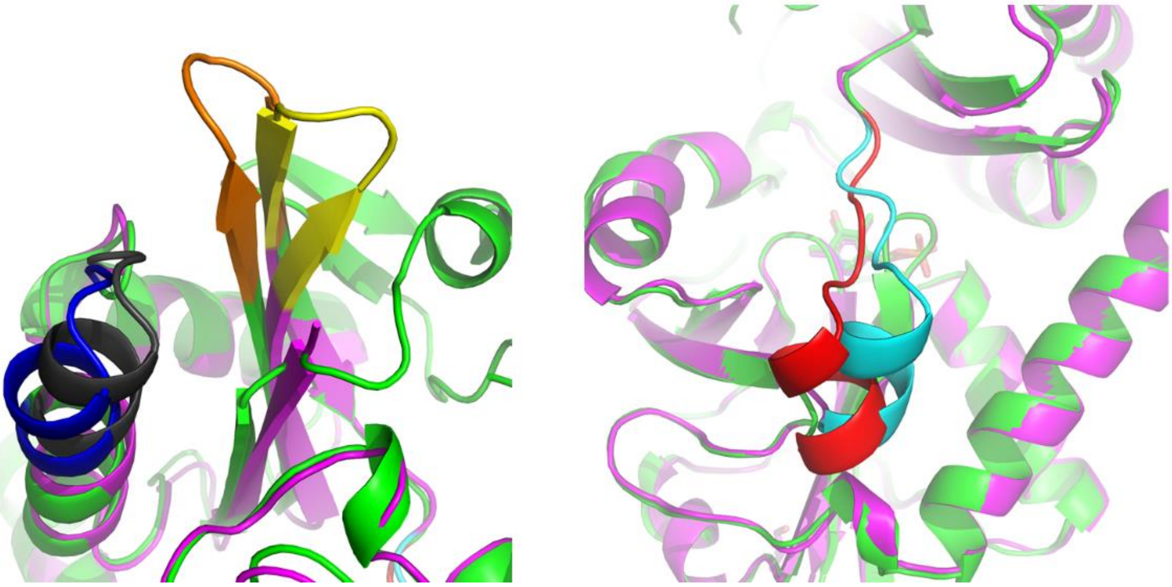

**Figure S2.** Superposition of *Dtol*TA (low resolution, green) and *Dtol*TA (high resolution, magenta). Residues 56-64, 95-102 and 110-120 are shown as blue, orange and red, respectively, in *Dtol*TA (low resolution) and grey, yellow and cyan, respectively, in *Dtol*TA (high resolution).

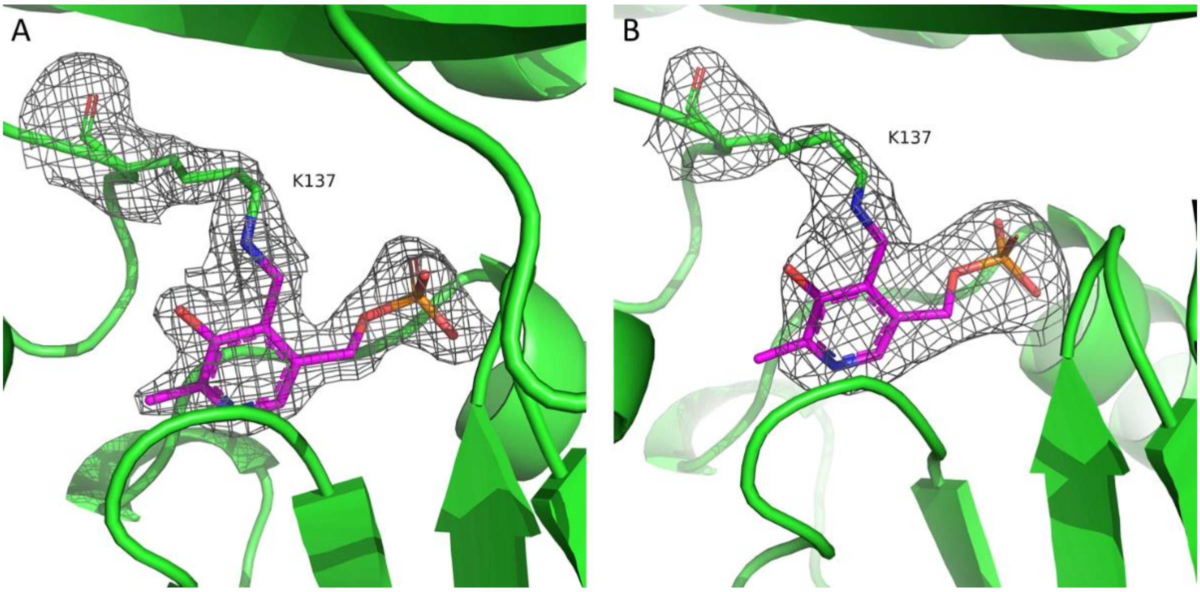


**Figure S3.** The omit map (Fo-Fc, grey mesh at 3σ level) of PLP covalently bound with K137 in (**A**) *Dtol*TA (high resolution) and (**B**) *Dtol*TA (low resolution). PLP is colored in magenta.

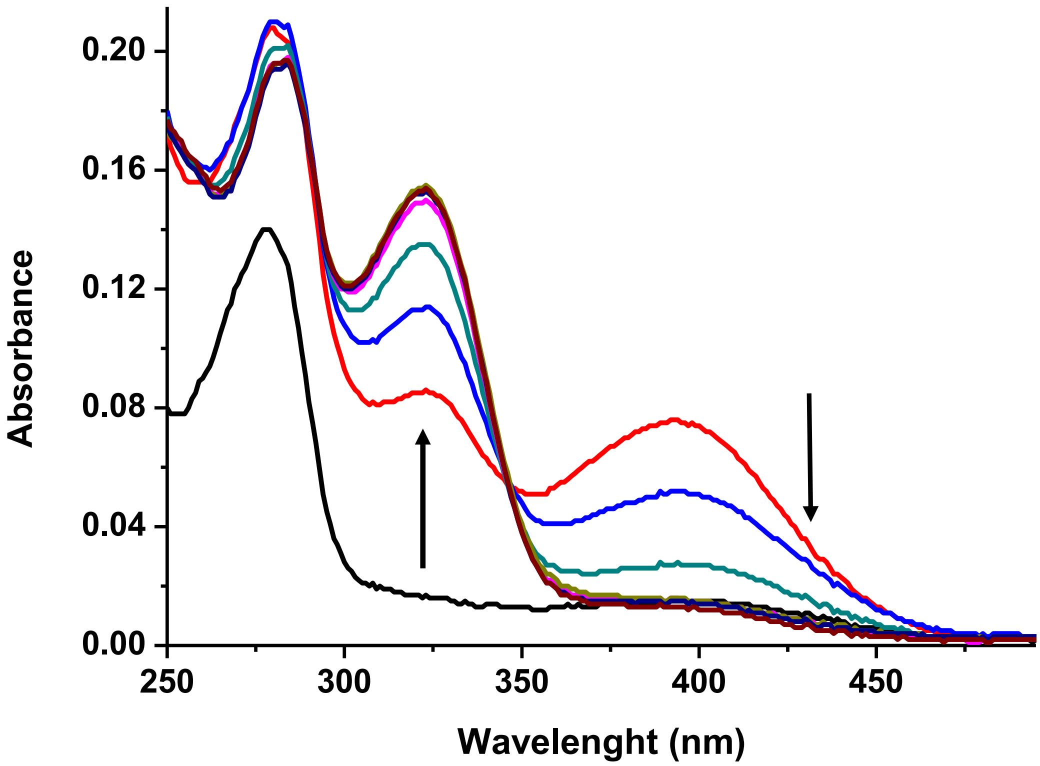


**Figure S4.** Half-reaction between the K91A variant (12.5 μM), 20 μM PLP and 10 mM D-alanine, spectrum of the apoenzyme is colored in black, the PLP decrease is observed at 390-400 nm, the PMP production at 324 nm. Reaction progress is shown by allows.

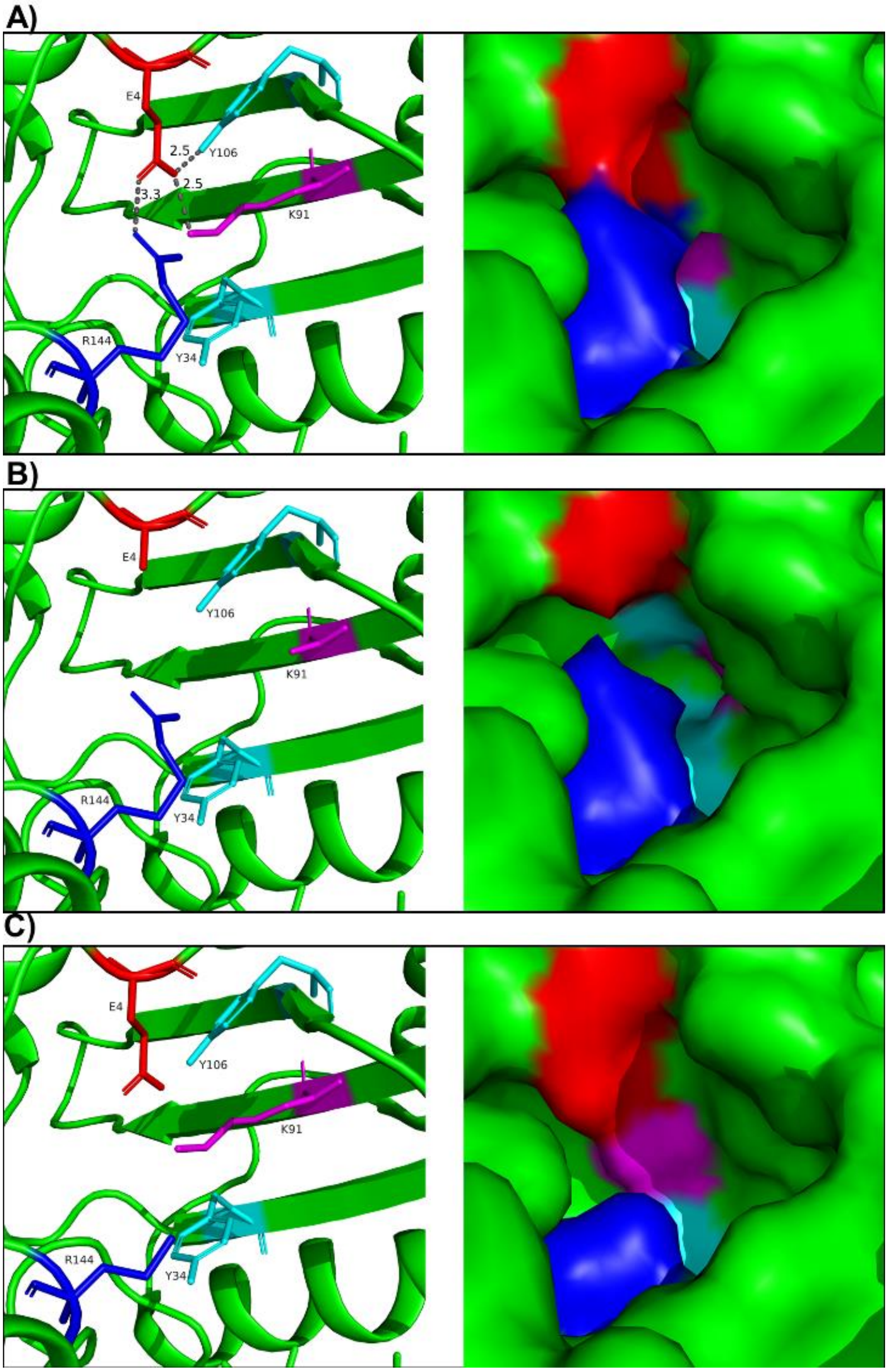


**Figure S5**. Structures and surfaces of the O-pocket region in (**A**) WT *Dtol*TA, **(B**) *Dtol*TA without sidechains of K91 (substitution K91A) and E4 and (**C**) *Dtol*TA without side chain of R144 (substitution R144I). Residues E4, K91 and R144 are colored in red, magenta and blue, respectively. Residues Y34 and Y106 are colored in cyan. Grey dotted lines show polar contacts, correspondent distances are in angstroms.

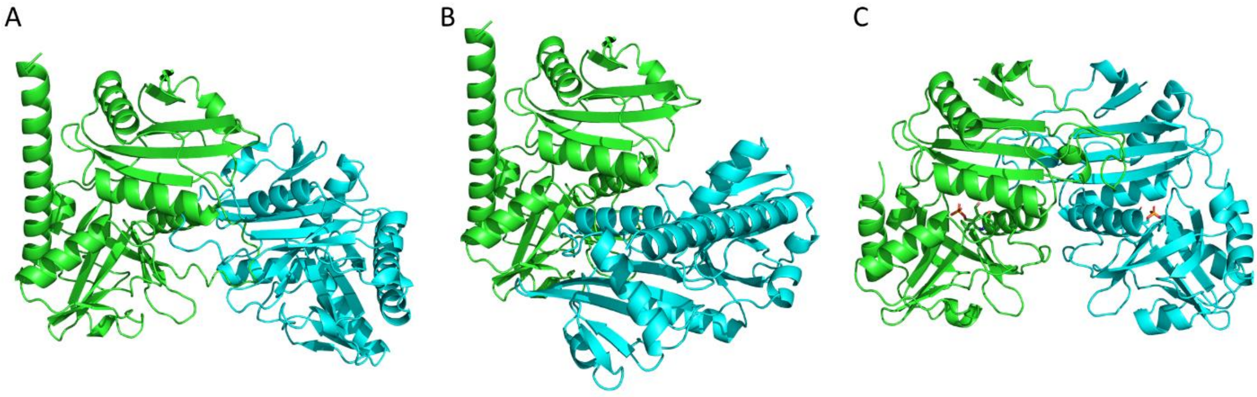


**Figure S6**. (**A**,**B**) Dimers of *Dtol*TA (low resolution) predicted by AlphaFold3. (**C**) Functional dimer of *bs*DATA (PDB ID: 1DAA). Green subunits on all panels are shown in the same orientation.

## References

1. Höhne, M.; Schätzle, S.; Jochens, H.; Robins, K.; Bornscheuer, U.T. Rational assignment of key motifs for function guides in silico enzyme identification. *Nat. Chem. Biol.* **2010**, *6*, 807–813, doi:10.1038/nchembio.447.
